# Osteogenic Properties of 3D-Printed Silica-Carbon-Calcite Composite Scaffolds: Novel Approach for Personalized Bone Tissue Regeneration

**DOI:** 10.3390/ijms22020475

**Published:** 2021-01-06

**Authors:** Parastoo Memarian, Francesco Sartor, Enrico Bernardo, Hamada Elsayed, Batur Ercan, Lucia Gemma Delogu, Barbara Zavan, Maurizio Isola

**Affiliations:** 1 Department of Animal Medicine, Productions and Health, University of Padova, 35020 Legnaro, Italy; parastoo.memarian@studenti.unipd.it (P.M.); francesco.sartor.10@studenti.unipd.it (F.S.); maurizio.isola@unipd.it (M.I.); 2 Department of Industrial Engineering, University of Padova, 35131 Padova, Italy; enrico.bernardo@unipd.it (E.B.); Hamada.Elsayed@unipd.it (H.E.); 3 Refractories, Ceramics and Building Materials Department, National Research Centre, El-Bohous Str., 12622 Cairo, Egypt; 4 Department of Metallurgical and Materials Engineering, Middle East Technical University, 06800 Ankara, Turkey; baercan@metu.edu.tr; 5 Biomedical Engineering Program, Middle East Technical University, 06800 Ankara, Turkey; 6 BIOMATEN, METU Center of Excellence in Biomaterials and Tissue Engineering, 06800 Ankara, Turkey; 7 Dipartimento di Scienze Biomediche, Università di Padova, 35100 Padova, Italy; LuciaGemma.delogu@unipd.it; 8 Department of Translational Medicine, University of Ferrara, 44121 Ferrara, Italy

**Keywords:** 3D printing, graphene, biomaterial

## Abstract

Carbon enriched bioceramic (C-Bio) scaffolds have recently shown exceptional results in terms of their biological and mechanical properties. The present study aims at assessing the ability of the C-Bio scaffolds to affect the commitment of canine adipose-derived mesenchymal stem cells (cAD-MSCs) and investigating the influence of carbon on cell proliferation and osteogenic differentiation of cAD-MSCs in vitro. The commitment of cAD-MSCs to an osteoblastic phenotype has been evaluated by expression of several osteogenic markers using real-time PCR. Biocompatibility analyses through 3-(4,5-dimethyl- thiazol-2-yl)-2,5-diphenyl tetrazolium bromide (MTT), lactate dehydrogenase (LDH) activity, hemolysis assay, and Ames test demonstrated excellent biocompatibility of both materials. A significant increase in the extracellular alkaline phosphatase (ALP) activity and expression of runt-related transcription factor (RUNX), ALP, osterix (OSX), and receptor activator of nuclear factor kappa-Β ligand (RANKL) genes was observed in C-Bio scaffolds compared to those without carbon (Bio). Scanning electron microscopy (SEM) demonstrated excellent cell attachment on both material surfaces; however, the cellular layer on C-Bio fibers exhibited an apparent secretome activity. Based on our findings, graphene can improve cell adhesion, growth, and osteogenic differentiation of cAD-MSCs in vitro. This study proposed carbon as an additive for a novel three-dimensional (3D)-printable biocompatible scaffold which could become the key structural material for bone tissue reconstruction.

## 1. Introduction

Substantial bone devitalization or loss caused by trauma, neoplasia, delayed union, nonunion, fixation of bone fractures, and corrective osteotomies are major unfulfilled demands in human and veterinary practices [1,2,3,4]. Nowadays, bone tissue engineering has emerged as a promising approach to restore bone defects by supporting bone tissue regeneration and reconstruction. Realizing the aim of bone tissue engineering is highly dependent on the functionality of the scaffold material [2,5,6,7]. Scaffolds are physical structures that serve as templates to support cell adherence, proliferation, and differentiation, and ultimately trigger tissue regeneration and regrowth. Ideally, the scaffolds for bone tissue engineering should be porous with good biocompatibility, controlled biodegradation, suitable material property, sufficient mechanical strength, and designed architecture to offer the potential applications for hard tissue repair [2,6,8].

In the past decade, three-dimensional (3D) printing has emerged as a versatile technology platform for the rapid manufacturing of synthetic substitutes for regenerative medicine. 3D printing is overtaking traditional machining and casting techniques for designing and manufacturing various biomedical devices. The benefits of 3D printing include not only the customization of medical products, equipment, and drugs, but also increased productivity, specificity, and cost-effectiveness [9]. In the biomedical field, 3D printing technologies are applied in (1) the creation of personalized prosthetics, implants, and anatomical models; (2) the reconstruction of organs and tissues; (3) the manufacturing of medical instruments [10]. Personalized implants are manufactured to fit the anatomy or other requirements of a specific single patient [5,10]. By monitoring several parameters of scaffold design, 3D printing technology can control the scaffold macrogeometry to perfectly adapt the implant to the tissue defect and the microarchitecture of the scaffold. In this manner, it could guarantee sufficient porosity and interconnectivity, as well as improve cell transportation and nutrient diffusion. Through an elevated degree of control, localization of biomolecular cues, and tailored mechanical properties, 3D printing can create complex material geometries that resemble endogenous tissues and exhibit analogous mechanical properties [5,11].

Nowadays, many synthetic and natural scaffolds have been investigated as bone graft substitutes for bone tissue engineering. Synthetic materials are profitable over natural materials for the simplicity of their manufacturing and customizable characteristics such as physical, biomechanical, and biochemical properties as well as cost-efficacy [2,7]. Recently, several materials have been introduced as promising scaffolds for the reconstruction or regeneration of orthopedic defects recognized as having excellent biocompatibility and biodegradability (polymeric scaffolds) [2] as well as great osteoinductivity and osteoconductivity (bioceramics and bioactive glasses) [1,10,12]. Although a number of these materials (e.g., polymers and bioceramics) have been considered for potential clinical applications, they sustained deficiencies to withstand the forces acting upon them. These poor mechanical properties including poor tensile strength and low fracture stiffness, limit their potential applications, especially when used under specific load-bearing forces [1,2,12]. To address these limitations, the carbon in the form of graphene and its derivatives, graphene oxide (GO) and reduced graphene oxide (rGO), can be exploited as specific nanoscale reinforcing fillers to fabricate graphene-based composites with increased overall mechanical properties [1,13,14,15]. 

Considering that the mechanical properties should influence the osteogenic commitment of stem cells, tissue engineering researchers started to use graphene for bone regeneration [13,14,16]. Bioceramics containing carbon, in the form of graphene or carbon nanotubes, were recently proposed and produced exceptional results in terms of biological and mechanical properties [6,15,17]. The use of silicone-based polymers ensures the possibility to form an “in-situ” carbon phase directly, attributable to the supervision of the ceramic modification managed by the firing atmosphere. In brief, silicones when heated in nitrogen form an amorphous silicon oxycarbide (SiOC) that is silica glass, with some Si-C bonds in the siloxanic network mixed with turbostratic carbon nanosheets [18]. Previously, we demonstrated that Si-C bonds did not form and exhibited a very remarkable strength improvement compared to C-free counterparts, with no degradation of biocompatibility [18].

Realizing the goal of bone tissue engineering to promote healing and regeneration of injured osseous tissue, stem cell-based bone tissue engineering employs 3D scaffolds for enhancing the regenerative capacity of stem cells [5,19]. In the of such considerations, the objectives of the present study were to assess the biocompatibility and morphology of a graphene-containing scaffold obtained with 3D printing technology and to investigate the influence of graphene on canine adipose-derived mesenchymal stem cells’ (cAD-MSCs) commitment. Therefore, the present study concentrated on the isolation and culture of canine adipose-derived mesenchymal stem cells (cAD-MSCs) and evaluating the cell behavior regarding the carbon-based scaffold material using additive manufacturing technology. Indeed, prior to being transferred into a canine orthopedic practice, it is crucial to evaluate the impacts of graphene on cell adhesion, proliferation, and differentiation of cAD-MSCs in vitro. This study will provide valuable outcomes regarding the potential application of graphene-based scaffold as an implantable scaffold for future studies of bone defects in small animal orthopedics.

## 2. Results

### 2.1. Biocompatibility and Cell Proliferation

#### 2.1.1. Lactate Dehydrogenase (LDH) and 3-(4,5-dimethyl- thiazol-2-yl)-2,5-Diphenyl Tetrazolium Bromide (MTT) Assays

**Figure 1:**
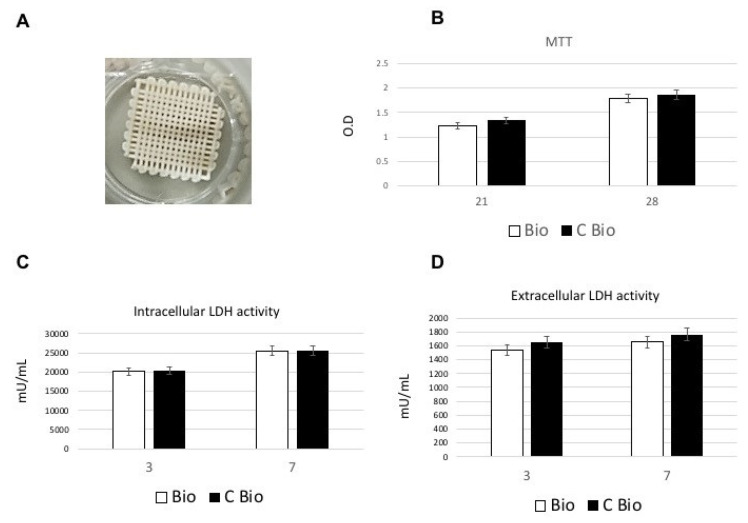
Biocompatibility analyses of scaffolds. (**A**) Image of a sample of the three-dimensional (3D)-printed scaffold used in the study. (**B**) 3-(4,5-dimethyl- thiazol-2-yl)-2,5-diphenyl tetrazolium bromide (MTT) assay of canine adipose-derived mesenchymal stem cells (cAD-MSCs) cultured on the Bio or C-Bio scaffolds for 21 and 28 days. The rate of proliferation of cAD-MSCs increased throughout the culturing period, reaching the highest rate after 28 days of culture. No significant differences were noted in terms of the proliferation rate among two types of scaffolds in these time points. (**C**,**D**) The toxicity of the surfaces was evaluated by lactate dehydrogenase (LDH) activity assay after 3 and 7 days of culture. No significant differences were observed among the two types of scaffolds at these time points. Data presented as mean ± standard error (3 measurements).

The cAD-MSCs were seeded and cultured on the 3D-printed scaffolds in the absence of carbon (Bio) (Figure 1A) or loaded with carbon (C-Bio) (Figure 1B) to assess the biocompatibility of these materials. MTT analysis was performed after 21 and 28 days of culture for measuring cell metabolic activity and as an indirect evaluation method for the cellular proliferation rate. Our results revealed that no significant differences in cell metabolic activity could be identified between the two types of scaffolds and the cells were proliferated favorably on the surfaces of the scaffolds, at these time points (Figure 1B). The toxicity of the surfaces can be evaluated by means of damage to cells, so it was evaluated by LDH activity assay. The intracellular level and extracellular levels of LDH have been reported in Figure 1C,D, respectively. As presented in Figure 1C, the cAD-MSCs were able to produce metabolites when seeded on both scaffolds’ surfaces, with superior outcomes following 7 days from seeding. The low LDH activity detected in the culture medium confirms that there was no damage due to the disruption of the membrane (Figure 1C). Moreover, no significant difference between the two scaffolds was observed (Figure 1C,D).

#### 2.1.2. Hemolysis Assay

An additional test aimed to evaluate the scaffold’s blood compatibility is the hemolysis assay. This test is usually applied for biomaterials which are mainly intended for blood-contacting applications. The results showed that the hemolysis index (HI) was less than 2% for both Bio and C-Bio scaffolds, indicating the absence of any hemolytic activity of the tested materials (Table 1).

#### 2.1.3. Ames Test

The Ames test was performed to evaluate the genotoxic ability of Bio and C-Bio bioceramics—namely, the ability of scaffolds’ materials to induce mutation on DNA. Four different strains of *Salmonella typhimurium* (STDisc™ TA1535, TA1537, TA98, TA100) were used in the bacterial mutagenesis assay. As reported in Table 2, no mutation was observed—neither in calcium phosphate (CaP) nor in graphene-calcium phosphate (G-CaP) scaffolds in any of the four different bacteria strains used.

### 2.2. Osteogenic Commitment

**Figure 2:**
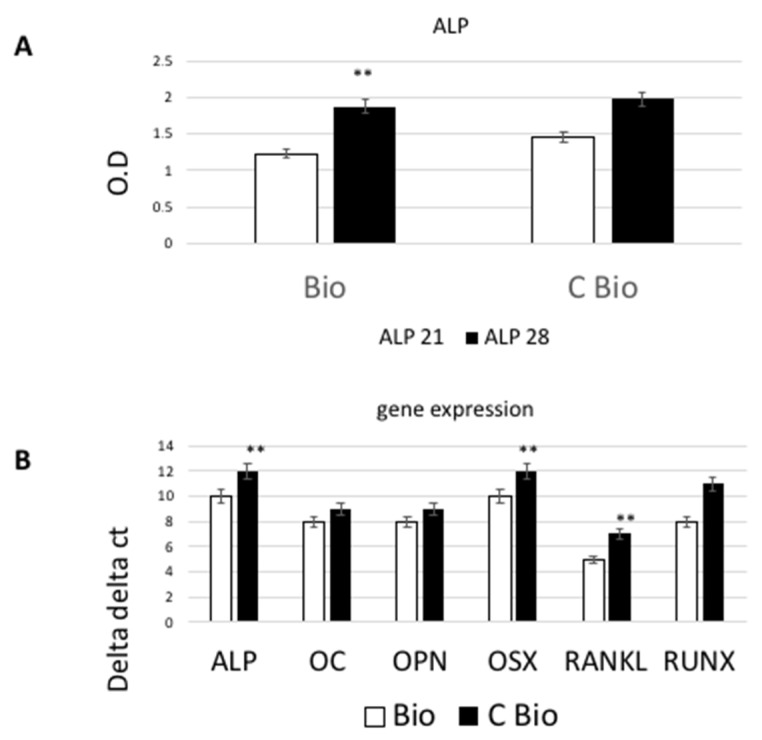
Alkaline phosphatase (ALP) production level and gene expression profiles of cAD-MSCs seeded on Bio and C-Bio scaffolds. (**A**) The extracellular ALP activity level of cAD-MSCs was higher in the graphene-loaded scaffolds than the controls at 21 and 28 days after cell culture. (**B**) The expressions of runt-related transcription factor (RUNX), ALP, osterix (OSX), and receptor activator of nuclear factor kappa-Β ligand (RANKL) genes were found to be increased significantly in the C-Bio surface compared to those of CaP, after 28 days of culture. Data presented as mean ± standard error (3 measurements). Statistically significant differences (*p* < 0.05) between G-CaP and CaP at a given day are indicated by the ** symbols.

To evaluate the differentiation process of cAD-MSCs toward the osteoblastic lineage in both scaffolds (with and without carbon), the expression of osteoblastic markers at late stages were analyzed through real-time RT-PCR. The stem cells’ commitment to an osteoblastic phenotype was evaluated by investigating the expression of several osteogenic markers, such as alkaline phosphatase (ALP) activity, osteocalcin (OC), osteopontin (OPN), osterix (OSX), receptor activator of nuclear factor kappa-Β (NFκB) ligand (RANKL), runt-related transcription factor (RUNX) using biochemical (for ALP) and RT-PCR after 28 days of cultures. Tests were performed on cells seeded on Bio or C-Bio scaffolds. To verify the primary cell differentiation of cAD-MSCs towards osteoblastic phenotype, the ALP activity (expressed as U/mL which is the quantity of enzyme contributing to the hydrolysis of one μmole of p-nitrophenyl phosphate (pNPP) per unit per mL) was measured in all cells condition. As reported in Figure 2A, the extracellular ALP was significantly higher in the graphene-loaded scaffolds as compared to the controls, at 21 and 28 days after culture (*p* < 0.05). The expressions of ALP, OSX, RANKL, RUNX genes were found to be augmented significantly (*p* < 0.05) in the graphene surface compared to the control after 28 days of culture (Figure 2B). On the other hand, OPN and OC gene expression levels did not show a significant difference between test and control surfaces.

### 2.3. Scanning Electron Microscopy (SEM) Analysis

**Figure 3:**
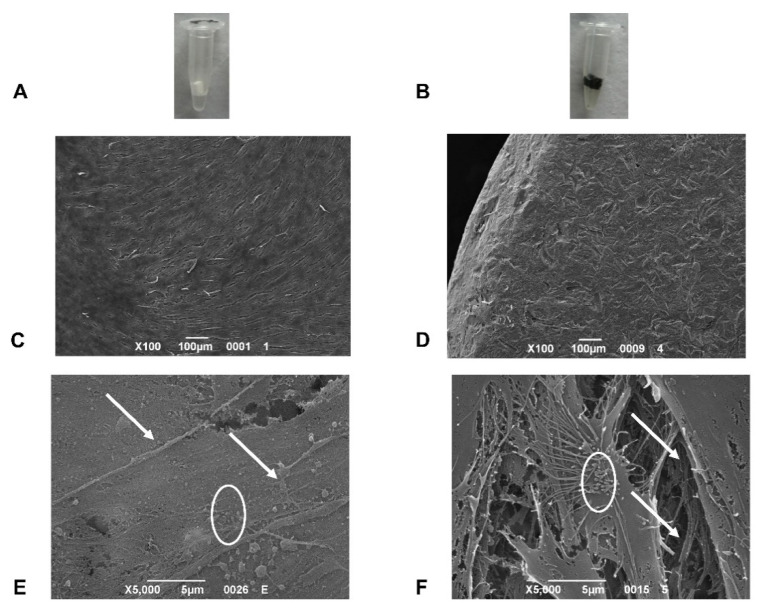
(**A**) BioC. (**B**) C-Bio. (**C**) SEM images of cAD-MSCs 7 days after culture on Bio scaffolds (Magnification 100×). (**D**) SEM images of cAD-MSCs 7 days after culture on C-Bio scaffolds (Magnification 100×). (**E**) SEM images of cAD-MSCs 7 days after culture on Bio scaffolds (Magnification 5000×). (**F**) SEM images of cAD-MSCs 7 days after culture on C-Bio scaffolds (Magnification 5000×). On both scaffolds’ surfaces, cells show visible filopodia (arrows) contributed to the cell attachment. The cell surfaces on C-Bio fibers reveal a superior secretome activity (circles) consisting of light granules of the extracellular matrix.

To examine the surface cell topographies of control and carbon-based bioceramic scaffolds, SEM analysis was performed 7 days after cell culture (Figure 3). Based on SEM images, the seeding cAD-MSCs were able to spread on both scaffold surfaces and into both fiber types. The cells were widely distributed and given rise to produce a uniform monolayer showing flat and star-like features corresponding to odontoblastic/osteoblastic-like cells (Figure 3C,D). On both scaffolds’ surfaces, cells displayed filopodia with thin cytoplasmic projections that extended beyond the leading margins of the cells suggesting the contribution to the cell attachments (Figure 3E,F). The cell surfaces on carbon-loaded fibers showed apparent secretome activity (Figure 3F), comprised of light granules of the extracellular matrix [20].

## 3. Discussion

In the past decade, 3D printing has been considered as a particularly valid additive manufacturing approach for constructing porous biocompatible scaffolds that can be customized to meet the specific patient needs, in addition to optimizing proliferation and differentiation of stem cells [6,8]. The implication of scaffold-based tissue engineering is based on the architecture and interconnected porosity of the scaffold material which contributes to a condition for bioactivity and osteoconductivity [2,4,5,6,8]. Although, porosity is also the major drawback of several scaffolds, accountable for its fragility and poor mechanical property which limits its application for replacing the bone tissues. Calcium phosphate scaffolds do not have adequate osteoinductive ability and their crystallinity and pore size are the other two factors that could affect their mechanical strength and flexural [1,19,21]. To maintain the stiffness of the scaffold material as well as its relatively high porosity and large grain size, carbon fiber and glass fiber have been added to this bioactive ceramic as additives to fabricate new hybrid carbon-based composites with enhanced mechanical strength [1,14,22].

Recently, several studies verified the excellent biocompatibility of carbon derivates such as graphene [14,17,23,24,25,26] as well as their capability to induce differentiation of stem cells into specific lineages. Graphene-coated materials, GO, and 3D graphene foam have shown the capability to improve cell viability and proliferation as well as to induce osteogenic differentiation of stem cells as compared to traditional scaffolds or substrates [2,14,23]. In the present study, we used carbon as an additive material to be incorporated into scaffold materials, to improve the biological properties and osteogenic capacity of the bioceramic composite. In our survey, Bio and C-Bio scaffolds were cocultured with cAD-MSCs. The biocompatibility analyses of scaffold materials by means of MTT, LDH activity, hemolysis analyses, and Ames test revealed excellent cytocompatibility, hemocompatibility, and no mutagenic activity of both scaffolds’ materials. Consistent with these findings, the results of the MTT assay demonstrated good cell viability along with the proliferation of cAD-MSCs on both Bio and C-bio scaffolds. Further in-depth preclinical studies are needed to find the ideal biomaterial with proper biological and mechanical properties, for bone tissue engineering in veterinary practice.

## 4. Materials and Methods

### 4.1. Scaffolds

Bio and C-Bio scaffolds were obtained following the protocol that we performed in our previous paper (Hamada et al. [18]).

### 4.2. Isolation and Culture of cAD-MSCs 

Canine fat specimens (10 mL) were collected from the suprascapular or interscapular region of healthy dogs using aseptic techniques and general anesthesia to isolate cAD-MSCs. The study protocol (5 March 2009; 20150) was approved by the Ethical Committee of the University of Padova 2005. Briefly, after washing with PBS (EuroClone, Milan, Italy), the tissue was minced and digested with 0.075% collagenase type II (Sigma-Aldrich, Saint Louis, MO, USA) for 3 h at room temperature while shaking continuously. Cells were pelleted and maintained at 37 °C and 5% CO2 in basal medium, (Dulbecco’s modified Eagle’s medium (DMEM) high glucose (EuroClone) with the addition of 10% fetal bovine serum (FBS; EuroClone), and 1% penicillin/streptomycin (EuroClone)). Cells were seeded onto the CaP or G-CaP scaffolds at a density of 5 × 10^6^ cells/cm^3^.

### 4.3. MTT Assay

After 21 and 28 days of culture, the MTT assay (Sigma Aldrich) was used to evaluate the metabolic activities of the cAD-MSCs on CaP or G-CaP scaffolds, based on the capability of viable cells to lessen MTT into a colored formazan product. On days 21 and 28, the medium was substituted with a medium consisting of 0.5 mg/mL MTT and was subsequently probed with cells for 5 h at 37 °C. For solubilizing salts, the cultures were incubated at 37 °C for 30 min in a solution of dimethylsulfoxide (DMSO). Spectrophotometric reading was performed at OD of 570 nm using the Anthos 2010 96 spectrophotometer (Anthos Labtec Instruments, Salzburg, Austria). The values obtained in the absence of cells were considered as background.

### 4.4. LDH Activity 

LDH activity was detected at 3 and 7 days after cell culture using a specific LDH Activity Assay Kit (Sigma-Aldrich). After cell lysis, the intracellular LDH activity was estimated. Each sample was incubated with a reaction mixture, and the resulting product was measured at OD of 450 nm using the Victor 3 plate reader.

### 4.5. ALP Activity Measurements 

The ALP activity was detected after 21 and 28 days of cell culture to evaluate the initial differentiation of cAD-MSCs into preosteoblasts. The colorimetric Abcam’s Alkaline phosphates kit (Abcam, Cambridge, UK) was utilized to identify the intracellular and extracellular ALP activities.

### 4.6. Ames Test

The mutagenic potential of scaffolds’ materials was assessed using the Salmonella mutagenicity complete test kit (Moltox, Molecular toxicology Inc., Boone, NC, USA) for the Ames test. The test was performed with four different bacteria strains and was replicated three times for each sample. 

### 4.7. Hemolysis Assay

The hemocompatibility of scaffolds was assessed by the hemolysis assay performed according to standard practices outlined in ASTM F756. The scaffolds were considered as nonhemolytic if the HI was ≤2%. The procedure was accomplished using the following formula for calculating the HI, applying the mean absorbance value (OD) for each group:

(1) $$\mathrm{HI}\;(\%)\;=\;\frac{\mathrm{OD}\;\left(\mathrm{test}\;\mathrm{material}\right)-\mathrm{OD}\;\left(\mathrm{negative}\;\mathrm{control}\right)}{\mathrm{OD}\;\left(\mathrm{positive}\;\mathrm{control}\right)-\mathrm{OD}\;\left(\mathrm{negative}\;\mathrm{control}\right)}\times 100$$

### 4.8. SEM Analysis

For SEM analysis, CaP and G-CaP scaffolds were fixed in 1% glutaraldehyde in 0.1 M phosphate buffer at 4 °C for 30 min. Fixed samples were washed in 0.1 M phosphate buffer and then dehydrated in ascending graded series of ethanol and finally dried in hexamethyldisilazane. The analysis was carried out with the SEM JEOL JSM-6490 (JEOL, Tokyo, Japan). 

### 4.9. Gene Expression

Total RNA was isolated from canine cells after 21 or 28 days of differentiation with a Total RNA Purification Plus Kit (Norgen Biotek Corporation, Thorold, ON, Canada). The RNAs were evaluated with a NanoDropTM ND-1000 (Thermo Fisher Scientific, Berlin Germany). In total, 500 ng of total RNA of each sample was used for the cDNA synthesis (SensiFASTTM cDNA Synthesis kit Bioline GmbH, Berlin, Germany) using a LifePro Thermal Cycler (Bioer Technology, Wuhan, China). The RT-PCR was carried out using SensiFASTTM SYBR No-ROX mix (Bioline GmbH) on a Rotor-Gene 3000 (Corbett Research, Sydney, Australia). Data analysis was performed using the ∆∆Ct method. Results were reported as fold regulation of target genes in the test group compared with the control group.

### 4.10. Statistical Analyses

The mean values for quantitative data were compared applying the nonparametric Kruskal–Wallis test for RT-PCR results. T-tests were used to determine significant differences (*p* < 0.05). Repeatability was calculated as the standard deviation (SD) of the difference between measurements. All analyses were carried out using SPSS 16.0 software (SPSS Inc., Chicago, IL, USA) (license of the University of Padua, Padova, Italy). Each test was performed on five different implants for each time point and repeated three times.

## 5. Conclusions

In the present study, we combined carbon with bioceramics to fabricate a 3D printable scaffold with superior characteristics for bone application. The carbon-based scaffold was cocultured with cAD-MSCs and the results of RT-PCR revealed a significant rise in the extracellular ALP activity and expression of RUNX, ALP, OSX, and RANKL osteogenic markers on the scaffold surfaces containing graphene in comparison to the CaP scaffold. In vitro biocompatibility evaluation of the scaffolds confirmed good cytocompatibility and hemocompatibility of both scaffold materials. Further observation by electron microscopy verified the wide expansion of cells into the material surfaces and fibers and the cells displayed excellent cell attachment and apparent secretome activity on the surface of graphene-loaded scaffolds. Overall, based on our results, graphene can improve cell osteogenic commitment, stimulating osteogenic differentiation of cAD-MSCs in vitro. The results of this study encourage the use of graphene-based scaffolds as innovative materials for bone tissue engineering in veterinary practice due to their excellent biocompatibility and osteoinductivity. Indeed, prior to translating these preclinical findings into veterinary orthopedic applications, the underlying mechanisms for bone cell responses to different materials and surface topographies should be investigated.

## Figures and Tables

**Table 1:** Results of the hemolysis assay.

Sample	OD	HI	Results
PC	0.91 ± 0.008	100%	Hemolytic
Bio	0.0126 ± 0.003	0%	Nonhemolytic
C-Bio	0.0125 ± 0.002	0%	Nonhemolytic

OD (optical density): absorbance value at 540 nm, mean of three independent experiments ± standard deviation (SD), HI: hemolysis index, PC: positive control (Sterile Water), Bio: bioceramic-based scaffold, C-Bio: carbon-based bioceramic scaffold.

**Table 2:** Results of the Ames test.

Sample	STDisc™ TA1535		STDisc™ TA1537		STDisc™ TA98		STDisc™ TA100	
	Rev/Plate ^a^	Result	Rev/Plate ^a^	Result	Rev/Plate ^a^	Result	Rev/Plate ^a^	Result
blank	4 ± 3	not mutagenic	5 ± 3	not mutagenic	5 ± 3	not mutagenic	3 ± 3	not mutagenic
NC ^b^	3 ± 2	not mutagenic	4 ± 2	not mutagenic	2 ± 2	not mutagenic	4 ± 2	not mutagenic
PC1 ^c^	945 ± 64	mutagenic	956 ± 94	mutagenic	932 ± 69	mutagenic	933 ± 81	mutagenic
PC2 ^d^	858 ± 54	mutagenic	874 ± 41	mutagenic	859 ± 43	mutagenic	862 ± 63	mutagenic
Bio ^e^	3 ± 2	not mutagenic	4 ± 2	not mutagenic	2 ± 2	not mutagenic	4 ± 2	not mutagenic
C-Bio ^f^	3 ± 2	not mutagenic	4 ± 2	not mutagenic	2 ± 2	not mutagenic	4 ± 2	not mutagenic

^a^ The number of revertants/plate: mean of three independent experiments ± SD, ^b^ NC: negative control (aluminum oxide ceramic rod), ^c^ PC1: positive control 1 (ICR 191 acridine), ^d^ PC2: positive control 2 (sodium azide), ^e^ Bio: tested sample (bioceramics), ^f^ C-Bio: tested sample (carbon-based bioceramics scaffold).

## Data Availability

Data is contained within the article.
